# Effects of First Diagnosed Diabetes Mellitus on Medical Visits and Medication Adherence in Korea

**Published:** 2018-02

**Authors:** KIM Hyeongsu, SHIN Soon-Ae, LEE Kunsei, PARK Jong-Heon, HAN Tae Hwa, PARK Minsu, Minsu Eunyoung, JEONG Hyoseon, LEE Jung-Hyun, AHN Hyemi, KIM Vitna

**Affiliations:** 1. Dept. of Preventive Medicine, School of Medicine, Konkuk University, Seoul, Korea; 2. Big Data Steering Dept., National Health Insurance Service, Seoul, Korea; 3. Dept. of Medical Engineering, College of Medicine, Yonsei University, Seoul, Korea; 4. Granduate School of Public Health, Inje University, Seoul, Korea; 5. Dept. of Public Health Administration, Hanyang Women’s University, Seoul, Korea

**Keywords:** Diabetes mellitus, Health screening, Medical visit, Medication possession ratio, Appropriate medical adherence

## Abstract

**Background:**

The National Health Insurance Service (NHIS) conducted a screening test to detect chronic diseases such as hypertension and diabetes in Korea. This study evaluated the effects of health screening for DM on pharmacological treatment.

**Methods:**

The data from qualification and the General Health Screening in 2012, the insurance claims of medical institutions from Jan 2009 to Dec 2014, and the diabetic case management program extracted from the NHIS administrative system were used. Total 16068 subjects were included. Visiting rate to medical institution, medication possession ratio and the rate of medication adherence of study subjects were used as the indices.

**Results:**

The visiting rates to medical institutions were 39.7%. The percentage who received a prescription for a diabetes mellitus medication from a doctor was 80.9%, the medication possession ratio was 70.8%, and the rate of medication adherence was 57.8%.

**Conclusion:**

The visiting rate, medication possession ratio and rate of medication adherence for DM medication were not high. In order to increase the visiting rate, medication possession ratio and rate of medication adherence, NHIS should support environment in which medical institutions and DM patients can do the role of each part.

## Introduction

Diabetes mellitus (DM) is a primary risk factor for myocardial infarction and stroke (1). The prevalence of DM among people who are more than 30 yr old in Korea increased from 8.6% in 2001 (males: 9.5%, females: 7.9%) to 11.0% in 2013 (males: 12.8%, females: 9.1%) (2). The death rate associated with DM in 2013 was 21.5 per 100000 people, ranked fifth among the 10 leading causes of death in Korea (3). Furthermore, in 2012, the medical insurance expenditures for patients with DM totaled approximately US $ 1.2 billion and accounted for 3.03% of all medical insurance costs (4). Despite the high social and economic burdens related to DM, the management levels for this disorder in 2013 were only a 74.3% recognition rate, a 65.9% treatment rate, and a 16.3% control rate. Significant gaps remain between the 2013 recognition and control rates and the objectives suggested by the third Korean national health promotion program, National Health Plan 2020, which suggests an 85% recognition rate, 65% treatment rate, and 35% control rate by 2020 (5). Therefore, the early diagnosis and treatment of DM are critical in order to increase the recognition, treatment, and control rates for DM.

In Korea, the National Health Insurance Service (NHIS) annually or biennially conducted a screening test, the General Health Screening (GHS) that aimed to detect chronic diseases such as hypertension or DM (6). The participation rate for the GHS increased from 43.2% in 2002 (5380998 examinees) to 72.9% in 2012 (11419350 examinees) (6). Evaluations on the GHS have been performed from the perspective of its effectiveness with cost (7–9) or without cost (10–13). Nevertheless, the development of GHS in terms of quality and quantity is achieved; the assessment for the treatment subsequent to early diagnosis through GHS remains uncertain (14).

There have been lots of studies on the medication adherence for DM and its related factors (15–22), but only a few studies about effect of first DM diagnosis on the medical visit for the early pharmacological treatment and medical adherence.

This study evaluated the effects of GHS for DM on early pharmacological treatment by investigating the visiting rates to medical institution, the medication possession rate (MPR), the rate of medication adherence (RMA) and their related factors among people firstly diagnosed with DM through the GHS in 2012 in Korea.

## Methods

**Fig. 1:**
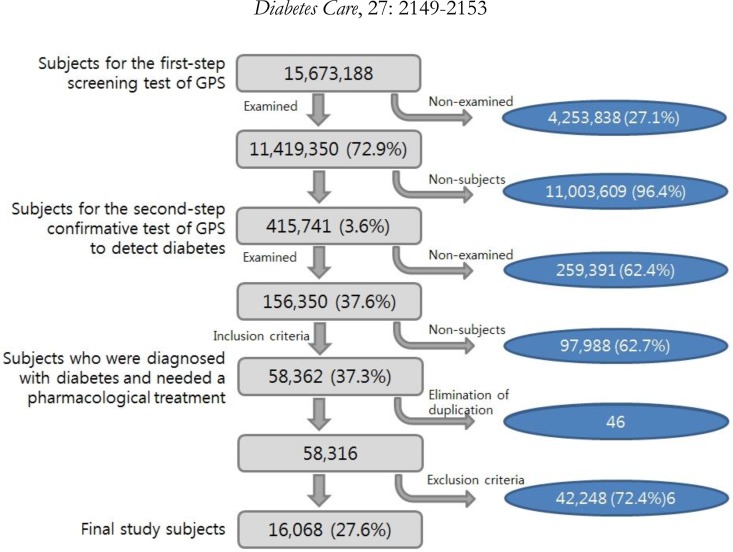
Selection process of the final study subjects

This study included the 2012 GHS’ participants diagnosed with DM and required pharmacological treatment simultaneously (Fig. 1).

The exclusion criteria were as follows: 1) participants who visited medical institutions due to hypertension, DM, or other related diseases as their principal or secondary diagnosis within the previous 3 yr of the date of the second-step confirmatory test, 2) participants with a history of a diagnosis and/or pharmacological treatment of hypertension, hyperlipidemia, myocardial infarction, or stroke based on the questionnaire of the first-step screening test, 3) participants who had even one instance of a fasting glucose level < 126 mg/dl at a first-step screening test and/or a second-step confirmatory test, and 4) participants who were under 30 yr of age at the time of the first-step screening test.

A total 15673188 individuals were the subjects for GHS in 2012 and 11419350 of them (72.9%) completed the first-step screening test. Of them, 415741 participants were subjects for the second-step confirmatory test of DM for having a fasting glucose level ≧126 mg/dl, and 156380 (37.6%) completed the second-step confirmatory test. Based on the results of the second-step confirmatory tests, 58362 participants were diagnosed with DM and also required pharmacological treatment. After 46 participants were excluded from the subject pool due to duplicate second-step confirmatory test results, 58316 participants remained. Of them, 39941 were excluded by the exclusion criterion. Thus, 16068 subjects were included in the analyses of this study.

This study analyzed the data from qualification and GHS in 2012, data from the insurance claims of medical institutions from Jan 2009 to Dec 2014, and data from the diabetic case management program extracted from the NHIS administrative system. The qualification data were used to determine gender (male/female), age, and type of insurance policy, case management program for diabetes. The GHS data were used to determine the family history of DM, smoking status, drinking frequency, obesity level, blood pressure levels, and blood glucose levels. The insurance claim data were used to determine the time of the first visit to a medical institution, hospitalization history, the number of outpatient clinic visits, and the number of prescription days for DM medication. The definition or explanations of variables or its subgroups were described in Table 1.

**Table 1:** Definition or explanation of variables or its subgroup

***Variable***	***Subgroups***	***Definition or explanation***
Gender		Male, Female
Age group(yr)		30–39, 40–49, 50–59, 60–69, ≧ 70
Family history of diabetes		Not present, Present
Smoking status		Nonsmoker, Ex-smoker, Smoker
Drinking frequency (per week)		Nondrinking, 1–2 times, ≧ 3 times
Types of insured		Self-employed, Employee, Medical aid
Obesity level	Normal	BMI < 23 kg/m^2^
	Overweight	23 kg/m^2^ ≤ BMI < 25 kg/m^2^
	Obese	25 kg/m^2^ ≤ BMI < 30 kg/m^2^
	Extremely obese	30 kg/m^2^ ≤ BMI
Blood pressure level	Normal	Systolic pressure : <120 mmHg and diastolic pressure : <80 mmHg
	Prehypertension	Systolic pressure : 120–139 mmHg or diastolic pressure : 80–89 mmHg
	Hypertension stage 1	Systolic pressure : 140–159 mmHg or diastolic pressure : 90–99 mmHg
	Hypertension stage 2	Systolic pressure : ≧ 160 mmHg or diastolic pressure : ≧ 100 mmHg
Blood glucose level	Mild	Blood glucose : 126–139 mg/dL
	Moderate	Blood glucose : 140–199 mg/dL
	Severe	Blood glucose ≧ 200 mg/dL
Time of the first visit to a medical institution (days)	Within 90	Time of the first visit to a medical institution during first one year from DM diagnosis
	91–180	
	After 181	
Hospitalization	No	Hospitalization or not for DM treatment from the first visit to a medical institution to the next one year
	Yes	
Number of the outpatient visits	1–2 times	Number of the outpatient visits during first one year from the first visit to a medical institution
	3–5 times	
	6–11 times	
	≧ 12 times	
Case management program for diabetes	Nonparticipants	Participation or not for case management program for DM during first one year from the first visit to a medical institution
	Participants	
Prescription days		Prescription days for DM medication during first one year from the first visit to a medical institution

BMI: Body mass index

### Measurement

1) Visiting rate to medical institution

A visit to a medical institution was used as an indicator of medical use. In this study, the rate of visiting a medical institution was defined as the percentage of DM patients who visited a medical institution as a principal or secondary diagnosis more than one time within 1 yr from the date after the DM diagnosis based on the result of the second-step confirmatory test:

$$\begin{array}{l} \text{Visiting}\;\text{rate}\;\text{to}\;\text{medical}\\ \text{institution} \end{array}\;=\;\frac{\begin{array}{l} \text{Number}\;\text{of}\;\text{DM}\;\text{patients}\;\text{who}\;\text{visited}\;\text{a}\;\text{medical}\;\text{institution}\\ \text{within}\;1\;\text{year}\;\text{from}\;\text{the}\;\text{date}\;\text{after}\;\text{diagnosis} \end{array}}{\text{Number}\;\text{of}\;\text{total}\;\text{study}\;\text{subjects}}\times 100$$

2) Medication Possession Ratio (MPR)

The MPR was defined as the percentage of the sum of the prescription days for a DM medication within one year from the first prescribed day among the subjects who visited a medical institution (15, 16).

$$\begin{array}{l} \text{Medication}\\ \text{Possession}\\ \text{Ratio} \end{array}\;=\;\frac{\text{Sum}\;\text{of}\;\text{the}\;\text{prescribed}\;\text{days}\;\text{within}\;\text{the}\;\text{time}\;\text{period}\;\text{of}\;\text{denominator}}{\text{One}\;\text{year}\;\text{from}\;\text{the}\;\text{first}\;\text{prescribed}\;\text{day}\;(365\;\text{d})}\times 100$$

When the MPR was greater than 100%, it was revised to be 100%.

3) Rate of Medication Adherence (RMA)

Medication adherence was defined as the values of MPR greater than 80% (17, 18). The RMA was calculated as the percentage of subjects who had MPR greater than 80% among people who received a prescription for DM.

$$\begin{array}{l} \text{Rate}\;\text{of}\;\text{Medication}\\ \text{Adherence} \end{array}\;=\;\frac{\text{Number}\;\text{of}\;\text{patients}\;\text{with}\;\text{MPR}\;\text{of}\;≧\;80\%}{\text{People}\;\text{who}\;\text{received}\;\text{the}\;\text{DM}\;\text{prescription}}\times 100$$

### Data analysis

All statistical analyses were conducted with SAS software (version 9.1; SAS Institute Inc., Cary, NC, USA). A chi-square analysis, t-test, or analysis of variance was used for comparison within subgroups of variables on the rate of visiting a medical institution, the MPR, and the RMA. Next, a multivariable logistic regression analysis was performed to identify variables significantly related to visiting a medical institution and RMA. The odds ratios (OR) and 95% confidence intervals (CI) of visiting rate were calculated. A *P-*value<0.05 was considered to indicate statistical significance.

### Ethics

This study was reviewed and approved by the Institutional Review Boards of Konkuk University Hospital (approval number: KUH1260021).

## Results

The visiting rates to medical institutions were 39.7% (n=6,377) for the total population, 37.0% for males, and 50.9% for females (*P*<0.001) (Table 2).

**Table 2:** Visiting rates to medical institutions and its related factors according to multiple logistic regression analysis

***Variables***		***Medical institution***		***Total (%)***	***P-value***	***Visit to medical institution***	
		**Visiting (%)**	**Nonvisiting (%)**			**OR**	**95% CI**
Total		6377 (39.7)	9691 (60.3)	16,068 (100)			
Gender	Male	4791 (37.0)	8162 (63.0)	12953 (80.6)	0.001	1	
	Female	1586 (50.9)	1529 (49.1)	3115 (19.4)		1.51	1.40–1.72
Age group	30–39	930 (30.8)	2086 (69.2)	3016 (18.8)	0.001	1	
	40–49	2237 (37.1)	3793 (62.9)	6030 (37.5)		1.31	1.19–1.45
	50–59	2230 (43.5)	2901 (56.5)	5131 (31.9)		1.81	1.63–2.01
	60–69	781 (50.7)	760 (49.3)	1541 (9.60)		2.38	2.07–2.74
	≥ 70	199 (56.9)	151 (43.1)	350 (2.20)		3.13	2.46–3.98
Family history of DM	Not present	3965 (37.6)	6580 (62.4)	10545 (79.7)	0.001	1	
	Present	1198 (44.6)	1489 (55.4)	2687 (20.3)		1.37	1.26–1.50
Smoking status	Nonsmoker	2475 (44.1)	3138 (55.9)	5613 (35.0)	0.001	1.02	0.93–1.12
	Ex-smoker	1422 (41.7)	1990 (58.3)	3412 (21.2)		1.28	1.17–1.40
	Smoker	2479 (35.2)	4558 (64.8)	7037 (43.8)		1	
Drinkingfrequency(per week)	Nondrinking	2450 (45.8)	2896 (54.2)	5346 (33.3)	0.001	1.30	1.18–1.44
	1–2 times	2545 (37.5)	4242 (62.5)	6787 (42.3)		1.17	1.08–1.28
	≧ 3 times	1379 (35.1)	2550 (64.9)	3929 (24.5)		1	
Types of insured	Self-employed	1267 (51.8)	1180 (48.2)	2447 (15.2)	0.001	1.53	1.39–1.67
	Employee	5047 (37.4)	8466 (62.6)	13513 (84.1)		1	
	Medical aid	63 (58.3)	45 (41.7)	108 (0.70)		1.81	1.21–2.70
Obesity level	Normal	1379 (42.1)	1922 (57.9)	3319 (20.7)	0.001	1	
	Overweight	1601 (41.6)	2251 (58.4)	3852 (24.0)		1.05	0.95–1.16
	Obese	2825 (38.9)	4434 (61.1)	7259 (45.2)		1.00	0.92–1.10
	Extremely obese	554 (33.8)	1084 (66.2)	1638 (10.2)		0.88	0.77–1.00
Blood pressure level	Normal	1477 (44.5)	1845 (55.5)	3322 (20.7)	0.001	1	
	Prehypertension	3697 (39.8)	5600 (60.2)	9297 (57.9)		0.89	0.81–0.96
	Hypertension stage 1	830 (36.5)	1443 (63.5)	2273 (14.2)		0.73	0.65–0.82
	Hypertension stage 2	373 (31.7)	803 (68.3)	1176 (7.3)		0.62	0.54–0.72
Blood glucose level	Mild	1261 (29.2)	3065 (70.8)	4326 (26.9)	0.001	1	
	Moderate	3307 (39.8)	5001 (60.2)	8308 (51.7)		1.78	1.64–1.93
	Severe	1809 (52.7)	1625 (47.3)	3434 (21.4)		3.41	3.09–3.77

The ORs of a visit to a medical institution for diabetes treatment were 1.51 for females (95% CI: 1.40–1.72) with males as a reference and 1.31 for subjects in their 40s (95% CI: 1.19–1.45), 1.81 for in subjects in their 50s (95% CI: 1.63–2.01), 2.38 for subjects in their 60s (95% CI: 2.07–2.74), and 3.13 for subjects in their 70s or older (95% CI: 2.46–3.98) with subjects in their 30s as a reference. The ORs of a visit to a medical institution by subjects with a family history of diabetes was 1.37 (95% CI: 1.26–1.50) compared with subjects without history, who were ex-smokers was 1.28 (95% CI: 1.17–1.40) compared with smokers, and 1.30 (95% CI: 1.18–1.44) and 1.17 (95% CI: 1.08–1.28) for nondrinkers and subjects who drank once to twice a week, respectively, compared with subjects who drank more than three times a week. In terms of types of insured, the ORs of a visit to a medical institution was 1.53 (95% CI: 1.39–1.67) and 1.81 (95% CI: 1.21–2.70) for the self-employed group and the medical aid group, respectively, using the employee group as a reference. With respect to blood pressure, the ORs of a visit to a medical institution were 0.89 (95% CI: 0.81–0.96), 0.73 (95% CI: 0.65–0.82), and 0.62 (95% CI: 0.54–0.72) for the prehypertension group, hypertension stage 1 group, and hypertension stage 2 group, respectively, compared with the normal group. In terms of blood glucose levels, the ORs for a visit to a medical institution were 1.78 (95% CI: 1.64–1.93) and 3.41 (95% CI: 3.09–3.77) for the moderate group and the severe group, respectively, compared with the mild group.

Of the subjects who visited a medical institution, the percentage who received a prescription for a DM medication from a doctor was 80.9% (n=5195), their MPR was 70.8%, and the RMA was 57.8% (Table 3).

**Table 3:** Medication possession ratio (MPR) and rate medication adherence (RMA)

***Variables***		***Total (%)***	***MPR (SE)***	***P-value***	***RMA (No)***	***P-value***
Total		5195 (100.0)	70.8 (0.48)		57.8 (3001)	
Gender	Male	3861 (74.3)	69.0 (0.57)	0.001	55.2 (2130)	0.001
	Female	1334 (25.7)	76.0 (0.90)		65.3 (871)	
Age group(yr)	30–39	779 (15.0)	62.2 (1.23)	0.001	45.8 (357)	0.001
	40–49	1856 (35.7)	69.8 (0.80)		55.5 (1030)	
	50–59	1763 (33.9)	73.0 (0.82)		61.8 (1090)	
	60–69	638 (12.3)	76.5 (1.30)		65.7 (419)	
	≥ 70	159 (3.1)	75.9 (2.64)		66.0 (105)	
Family history of diabetes	Not present	3179 (76.2)	69.8 (0.62)	0.026	56.4 (1794)	0.045
	Present	993 (23.8)	72.9 (1.06)		60.4 (600)	
Smoking status	Nonsmoker	2008 (38.7)	73.2 (0.76)	0.001	61.7 (1238)	0.001
	Ex-smoker	1122 (21.6)	72.6 (1.02)		60.3 (677)	
	Smoker	2064 (39.7)	67.4 (0.78)		52.6 (1086)	
Drinking frequency (per week)	Non-drinking	2034 (39.2)	73.8 (0.75)	0.001	62.2 (1265)	0.001
	1–2 times	2045 (39.4)	69.1 (0.78)		55.3 (1130)	
	≥ 3 times	1113 (21.4)	68.3 (1.05)		54.3 (604)	
Types of insured	Self-employed	1049 (20.2)	72.0 (1.07)	0.377	60.4 (634)	0.138
	Employee	4092 (78.8)	70.5 (0.54)		57.1 (2335)	
	Medical aid	54 (1.0)	68.5 (5.17)		59.3 (32)	
Obesity level	Normal	1132 (21.8)	71.6 (1.02)	0.059	58.5 (662)	0.072
	Overweight	1303 (25.1)	71.7 (0.96)		59.4 (774)	
	Obese	2282 (44.0)	70.6 (0.73)		57.6 (1317)	
	Extremely obese	472 (9.1)	66.9 (1.65)		52.5 (248)	
Blood pressure level	Normal	1200 (23.1)	70.7 (0.98)	0.023	56.8 (682)	0.004
	Prehypertension	2996 (57.7)	69.8 (0.64)		56.4 (1691)	
	Hypertension stage 1	691 (13.3)	73.4 (1.31)		62.7 (433)	
	Hypertension stage 2	308 (5.9)	74.3 (1.92)		63.3 (195)	
Blood glucose level	Mild	816 (15.7)	68.8 (1.26)	0.016	56.3 (459)	0.351
	Moderate	2705 (52.1)	70.2 (0.68)		57.4 (1553)	
	Severe	1674 (32.2)	72.7 (0.81)		59.1 (989)	
Time of the first visit to a medical institution (days)	Within 90	3160 (60.8)	72.0 (0.61)	0.016	59.1 (1866)	0.050
	91–180	737 (14.2)	69.1 (1.31)		56.9 (419)	
	After 181	1298 (25.0)	68.6 (0.98)		55.2 (716)	
Hospitalization	No	4894 (94.2)	71.3 (0.50)	0.078	57.5 (2813)	0.090
	Yes	301 (5.8)	74.9 (1.95)		62.5 (188)	
Number of outpatient visits	1–2 times	772 (15.8)	34.7 (1.34)	0.004	22.5 (174)	0.001
	3–5 times	1077 (22.1)	57.6 (1.08)		38.2 (411)	
	6–11 times	1925 (39.4)	82.0 (0.55)		67.3 (1295)	
	≧12 times	1109 (22.7)	92.8 (0.44)		88.7 (984)	
Case management program for diabetes	Non-participants	4948 (95.2)	71.0 (2.30)	0.011	58.1 (2874)	0.038
	Participants	247 (4.8)	65.3 (0.49)		51.4 (127)	

SE : Standard error, No: Number

Based on the multivariable logistic regression analysis (Table 4), the OR of medication adherence were 1.17 for females (95% CI: 0.95–1.44) with males as a reference and 1.29 (95% CI: 1.05–1.59) for subjects in their 40s, 1.61 (95% CI: 1.30–2.01) for subjects in their 50s, and 1.68 (95% CI: 1.27–2.22) for subjects in their 60s using subjects in their 30s as a reference.

**Table 4:** Related factors of the rate of medication adherence according to multiple logistic regression analysis

		***Medication adherence***	
		**OR**	**95% CI**
Gender	Male	1	
	Female	1.17	0.95–1.44
Age group(yr)	30–39	1	
	40–49	1.29	1.05–1.59
	50–59	1.61	1.23–2.01
	60–69	1.68	1.27–2.22
	≥ 70	1.28	0.82–2.01
Family history of diabetes	Not present	1	
	Present	1.22	1.02–1.45
Smoking status	Nonsmoker	1.09	0.90–1.32
	Ex-smoker	1.18	0.99–1.42
	Smoker	1	
Drinking frequency (per week)	Nondrinking	1.12	0.91–1.36
	1–2 times	1.09	0.91–1.30
	≧ 3 times	1	
Types of insured	Self-employed	1.06	0.89–1.26
	Employee	1	
	Medical aid	1.24	0.60–2.58
Obesity level	Normal	1	
	Overweight	1.04	0.85–1.26
	Obese	1.05	0.88–1.26
	Extremely obese	0.84	0.64–1.104
Blood pressure level	Normal	1	
	Prehypertension	1.09	0.92–1.29
	Hypertension stage 1	1.45	1.14–1.85
	Hypertension stage 2	1.84	1.33–2.54
Blood glucose level	Mild	1	
	Moderate	1.06	0.87–1.28
	Severe	1.05	0.85–1.28
Time of the first visit to a medical institution (days)	Within 90	1	
	91–180	0.99	0.83–1.31
	After 181	1.02	0.87–1.17
Hospitalization	No	1	
	Yes	0.91	0.69–1.20
Number of the outpatient visits	1–2 times	0.02	0.01–0.02
	3–5 times	0.04	0.03–0.05
	6–11 times	0.22	0.18–0.27
	≧ 12 times	1	
Case management program for diabetes	Nonparticipants	1	
	Participants	0.87	0.64–1.18

Using subjects without a family history of diabetes as a reference, the OR of appropriate medication adherence for subjects with a family history of diabetes was 1.22 (95% CI: 1.02–1.45). In terms of blood pressure, the ORs of appropriate medication adherence was 1.09 (95% CI: 0.92–1.29), 1.45 (95% CI: 1.14–1.85), and 1.84 (95% CI: 1.33–2.54) for the prehypertension group, the hypertension stage 1 group, and the hypertension stage 2 group, respectively, compared with the normal group. In terms of the number of visits, the ORs of appropriate medication adherence were 0.02 (95% CI: 0.01–0.02), 0.04 (95% CI: 0.03–0.05), and 0.22 (95% CI: 0.18–0.27) for subjects who visited 1–2 times, 3–5 times, and 6–11 times, respectively, with subjects who visited more than 12 times as a reference.

## Discussion

This study aimed to determine the effects of GHS for DM on the early pharmacological treatment of Korea.

The primary purpose of health screening is the early identification of individuals with diseases, and this purpose is only fully accomplished when it leads to the initiation of early treatment. This study was the first attempt to evaluate the visiting rates to medical institutions for the pharmacological treatment among those diagnosed with DM by GHS. The visiting rate to a medical institution within one year of subjects diagnosed who were with DM was 39.7%, and the visitor’s median latency from diagnosis to visiting an institution was 48 d (data not shown). The National Breast and Cervical Cancer Early Detection Program in the US has an established standards requiring that women diagnosed with precancerous diseases or invasive cancers must initiate treatment within 60 d from the day of the final diagnosis. Moreover, the median times from diagnosis to treatment for invasive breast and cervical cancers are 14 and 21 d, respectively (23, 24). Furthermore, more than 90% of these women who receive complete diagnostic care initiate treatment in less than 30 d from the time of their diagnosis (25). In addition, the visiting rates to a medical institution were higher in females, older age groups, subjects with a family history of DM, ex-smokers, nondrinking subjects, subjects who drank less frequently, subjects with normal blood pressure levels, and subjects with higher blood glucose levels.

The percentage of subjects who visited a medical institution and prescribed DM medication at the same time was 80.9%. 19.1% of medical institution visitors who did not receive a prescription might be encouraged to manage their diabetic status through changing health behaviors and/or medical follow-ups rather than taking prescribed DM medication. Furthermore, the MPR and the RMA were 70.8% and 57.8% respectively. A study that analyzed the insurance claims data of 40082 individuals diagnosed with type 2 DM for the first time in outpatient clinics in 2004 found that the MPR for 2 yr of hypoglycemic agents was 49.5% and that the RMA was 29.3% (19). The higher MPR and RMA observed in this study suggest that treatment environments that require a more active participation of the DM patients may result in changes that are more positive. The RMA for oral hypoglycemic medications by diabetic patients ranged from 36% to 93% for those who remained in treatment for 6 to 24 months (16). Since each study and disease has its own definitions of the evaluation methods, medication adherence, and follow-up periods for medication adherence, it is not easy to compare the results among studies. In this study, the factors related to RMA included age, family history of DM, blood pressure, and the number of outpatient clinic visits. The RMA is higher in diabetics with comorbid diseases or in those who take more than two kinds of hypoglycemic agents (20, 21); patients are more likely to participate in medication treatment actively if the disease is more severe and they realize the importance of disease management. In this study, the subjects with higher blood pressure showed higher success rates in terms of MPR and RMA.

This study has several limitations. First, due to the use of secondary data from health screening and medical insurance claims, it was impossible to assess the subjects’ educational backgrounds or attitudes, the type or severity of DM, or the accessibility of the subjects to medical institutions. Second, because this study used a principal or secondary diagnosis code to identify the people with DM among the medical insurance claims data, diabetic patients may have been excluded from the analyses if DM was not their principal or secondary diagnosis, even if treatment was in progress. However, because subjects with a history of treatment for DM or related diseases were excluded from this study by the subject’s selection process, there is a low possibility that DM was not recorded as a principal or secondary diagnosis at the time of first treatment for DM. Third, this study used MPR and the number of DM medication’s prescription days to evaluate the medication adherence, but it is impossible to determine whether a patient actually purchased the medicine or ingested or not. However, the doctor’s way of prescription it is meaningful from the standpoint of patients; therefore, MPR can be considered a useful measuring tool for determining the RMA (15, 26). In addition, because it is almost impossible to ascertain the actual truth concerning medicine taking in retrospective research using large-scale databases, MPR is considered as the best option with which to evaluate medication adherence in general.

## Conclusion

Medical institutions should notify the DM patients about their health status, encourage, and educate them to participate actively in treatment. Next, DM patients should follow physician’s directions and cooperate with a physician to manage DM. NHIS should support environment in which medical institutions and DM patients can do the role of each part.

## Ethical considerations

Ethical issues (Including plagiarism, informed consent, misconduct, data fabrication and/or falsification, double publication and/or submission, redundancy, etc.) have been completely observed by the authors.
